# Evolution of “invasion syndrome” in invasive goldenrod is not constrained by genetic trade‐offs

**DOI:** 10.1111/eva.13734

**Published:** 2024-06-28

**Authors:** Laura C. Rigby, Matthew D. Hall, Keyne Monro, Akane Uesugi

**Affiliations:** ^1^ Biosciences and Food Technology RMIT University Bundoora Victoria Australia; ^2^ School of Biological Sciences Monash University Clayton Victoria Australia

**Keywords:** allelopathy, dispersal, EICA hypothesis, genetic (co)variance, G‐matrix, invasive species, partial diallel design, *Solidago altissima*

## Abstract

A suite of plant traits is thought to make weed populations highly invasive, including vigorous growth and reproduction, superior competitive ability, and high dispersal ability. Using a breeding design and a common garden experiment, we tested whether such an “invasion syndrome” has evolved in an invasive range of *Solidago altissima*, and whether the evolution is likely to be genetically constrained. We found an overall shift in invasive phenotypes between native North American and invasive Japanese populations. The invasive populations were taller and produced more leaves, suggesting a superior ability to exploit limited resources. The populations also produced more allelopathic compounds that can suppress competitor growth. Finally, invasive populations produced more seeds, which are smaller and are released from a greater height, indicating a potential for superior dispersal ability than the native populations. Quantitative genetics analyses found a large amount of additive genetic variation in most focal traits across native and invasive populations, with no systematic differences in its magnitude between the ranges. Genetic covariances among three traits representing invasion strategies (leaf mass, polyacetylene concentration and seed size) were small. The R metric, which measures the effect of genetic covariances on the rate of adaptation, indicated that the covariance neither constrains nor accelerates concerted evolution of these traits. The results suggest that the invasion syndrome in *S. altissima* has evolved in the novel range due to ample additive genetic variation, and relatively free from genetic trade‐offs.

## 1 INTRODUCTION

Invasive plants are often characterized as having superior ability to occupy available space, to out‐compete other plants within the community, and to disperse broadly across the landscape (Moran & Alexander, 2014; Richardson et al., 2000). Inter‐specific contrasts have identified a suite of plant traits that are associated with successful invasion (e.g., vigorous growth and reproduction, high resource acquisition capacity, prolific seed production, and high dispersal ability; Baker, 1965; Gallagher et al., 2014; Hamilton et al., 2005; Lloret et al., 2005; Pyšek & Richardson, 2007), suggesting the presence of an “invasion syndrome” across plant species (Lloret et al., 2005). Intra‐specific contrasts of native and invasive populations in common gardens further indicate that these invasive phenotypes could rapidly evolve in the novel range (Bossdorf et al., 2005; Colautti et al., 2009; Felker‐Quinn et al., 2013; Maron et al., 2004; Rotter & Holeski, 2018; Whitney & Gabler, 2008). But we have limited knowledge of how much genetic variance exists for these traits, and whether their evolution is genetically constrained, within the introduced range (McGoey & Stinchcombe, 2021).

Evolution of increased competitive ability (EICA; Blossey & Notzold, 1995) is predicted within plants' introduced ranges because a release from natural enemies should favor individuals that allocate more resources towards growth and competition in expense of reduced defense. Whereas tests of the EICA hypothesis generally focus on competitive traits that mediate resource exploitation (e.g., growth rate and biomass; Rotter & Holeski, 2018), some plants also compete by interfering with growth of neighbours through allelopathy (i.e., a release of toxic secondary metabolites into the environment). A related hypothesis, the novel weapons hypothesis (NWH; Callaway & Ridenour, 2004), suggests that allelopathy can be particularly effective in a species' novel range because indigenous competitors are naïve to the novel toxins. Thus, the combination of novel competitive environments and the release from herbivory may drive the evolution of enhanced allelopathy in the novel range (Callaway & Ridenour, 2004; Gruntman et al., 2016; Uesugi & Kessler, 2013, 2016). Among the limited studies that explored evolution of allelopathy, some showed enhanced allelopathy in invasive populations (Gruntman et al., 2016; Uesugi et al., 2020; Yuan et al., 2013; Zheng et al., 2015), while others showed no consistent changes (Bock et al., 2018; Irimia et al., 2019; Oduor et al., 2020; Sotes et al., 2021).

Invasive spread across the landscape further requires prolific production of seeds that are highly dispersive (Leishman et al., 2000; Lloret et al., 2005; Pergl et al., 2011). Seed traits that enhance dispersal (e.g., greater plant height, smaller seed size, and larger pappus; Soons et al., 2004; Saastamoinen et al., 2018) may be favored in introduced populations, particularly at the invasion front (Ochocki & Miller, 2017; Phillips et al., 2006). Evolution of dispersal ability at the invasion front was found in animal species (e.g., cane toads: Phillips et al., 2006; beetles: Ochocki & Miller, 2017) and some plants (Huang et al., 2015; Monty & Mahy, 2010; Tabassum & Leishman, 2018, but not in Bartle et al., 2013). In an experimental evolution study, Williams et al. (2016) showed that populations of *Arabidopsis thaliana* that evolved in more fragmented landscapes (i.e., greater distances between artificial habitat patches) were taller and dispersed more than control populations after six generations, indicating that dispersal ability could evolve rapidly.

Novel environments in an introduced range are expected to select for a combination of invasive traits, but whether these traits can respond to selection depends on the amount of additive genetic variation for the traits within the introduced populations (Lande, 1979). Evolutionary responses may be limited if genetic variation for the traits is reduced by founder effects (Dlugosch & Parker, 2008), although multiple introductions and genetic admixture are likely to supplement genetic diversity (Qiao et al., 2019; Roman & Darling, 2007). The amount of additive genetic variation may also differ among plant traits. For example, a meta‐analysis found that vegetative traits generally have greater genetic variance within populations compared to floral characteristics, and that the variance was positively correlated with the degree of population divergence in traits (Opedal et al., 2023). Thus, divergence of certain traits could be constrained by limited additive genetic variance.

Even with sufficient genetic variation for each trait, evolution of multiple traits could be constrained by genetic correlations among them (Blows & Walsh, 2009). Genetic trade‐offs among invasive traits may be expected: for example, resource investment for allelopathy may come at the cost of reduced growth if allelochemical production is energetically costly (Herms & Mattson, 1992). Dispersal ability that favors small seed size may also trade‐off with establishment probability and subsequent growth (Maron et al., 2021). However, we know little about the genetic architecture of these quantitative traits associated with invasiveness in introduced plant populations (Bacigalupe, 2009). Only a few studies have estimated additive genetic variances and covariances (summarised as a ***G***‐matrix) for a range of invasive traits in native and invasive populations, and they found small differences in the size and shape of ***G*** between the ranges (Calsbeek et al., 2011; Franks et al., 2012; McGoey & Stinchcombe, 2021; Sakata et al., 2020).

Here, we investigate whether multivariate invasive phenotypes of tall goldenrod, *Solidago altissima* L. (Asteraceae), are genetically differentiated between invasive Japanese populations and native North American populations. We explore 10 focal traits that are associated with plant invasiveness: Plants may enhance resource exploitation by growing faster, increase the specific leaf area (SLA), height and leaf biomass to shade out competitors, and produce more rhizomes to compete for space (Hamilton et al., 2005; Pyšek & Richardson, 2007; Root, 1996). Production of allelopathic polyacetylenes from roots would enhance interference competition (Uesugi et al., 2019). Early onset of flowering and longer duration of flowering period may result in greater seed production, while smaller seed size and height at which seeds are released should aid in long‐distance dispersal (Thomson et al., 2011; Williams et al., 2016).

Using a breeding design and a common garden experiment, we tested (1) if the 10 focal traits show divergence between native and invasive ranges, (2) if the amount of additive genetic variance present within a population for these traits varies between the ranges, and (3) whether the amount of genetic variance for each trait explains the degree of trait divergence between the ranges (Opedal et al., 2023). For a subset of traits that represent each of the three invasive strategies, and that consistently show significant levels of additive genetic variance (i.e., leaf mass for exploitative competition, polyacetylene concentrations for interference competition, and seed size for dispersal), we also tested (4) whether genetic (co)variance structure differs between native and invasive ranges, and (5) whether genetic covariance among these traits would constrain concerted evolution of these invasive traits.

## 2 METHODS

### 2.1 Study system

The tall goldenrod, *S. altissima* L. (Asteraceae), is a perennial forb native to eastern North America (Werner et al., 1980) and is a dominant species of old fields and disturbed habitats. *Solidago altissima* was first introduced into Japan in the late 1890s as an ornamental plant but only became widespread across the country since the 1980s (Fukuda, 1982). Current molecular data suggests that invasive Japanese *S. altissima* populations are likely to be introduced primarily from south‐eastern North America (Sakata et al., 2015), which may overlap with the distribution of a putative *S. altissima* variety, *pluricephala* that has also invaded other parts of Asia (Semple et al., 2015). The Japanese populations seem to be founded by multiple introduction events, resulting in similar levels of genetic diversity within a population at neutral markers as in the native populations (Sakata et al., 2015; Uesugi et al., 2020). Moreover, broad‐sense genetic variation for herbivore resistance traits did not differ between the US and Japanese populations (Sakata et al., 2020), suggesting that evolution in introduced Japanese populations may not be genetically constrained. However, additive genetic variation and covariation among traits associated with competitive and dispersal abilities has not been examined previously.

In its native range, a diverse group of specialist and generalist herbivores keeps the *S. altissima* populations in check (Root, 1996), whereas in the invaded Japanese range, the plant generally escapes herbivory, except from *Uroleucon nigrotuberculatum* (a *Solidago* specialist aphid introduced in 1990s, Cappuccino, 1987; Sugimoto & Matsumoto, 2000) and *Corythucha marmorata* (an Asteraceae specialist lacebug introduced to Japan in 2000, Kato & Ohbayashi, 2009; Sakata et al., 2014). Upon establishment in a field patch, *S. altissima* can rapidly grow tall and shade out neighbouring plants (Root, 1996), while suppressing germination and growth of competitor plants through allelopathy (Kobayashi et al., 1980; Johnson et al., 2010; Uesugi et al., 2019). It produces several compounds of polyacetylenes in roots, which are released into the soil at a concentration known to inhibit the growth of several other plant species (Kobayashi et al., 1980; Johnson et al., 2010; Uesugi & Kessler, 2013; Uesugi et al., 2019). Within a patch, *S. altissima* primarily spreads through rhizome production (Hartnett & Bazzaz, 1983), but colonization of new patch relies on wind‐born seeds that are produced in abundance (Uesugi et al., 2020).

### 2.2 Experimental design

#### 2.2.1 Seed sources and the quantitative genetic breeding design

Seeds were collected in 2016 from three native populations in the south‐eastern United States within the known range of *S. altissima* var. *pluricephala* where invasive Japanese populations are likely to have originated (Durham, NC, Spartanburg, SC, and Murrells Inlet, SC; Sakata et al., 2015; Table S1). Three invasive populations in Japan were sampled across a similar latitudinal range (Utsunomiya, Shizuoka, and Otsu). A principal component analysis (PCA) of variation in 19 WorldClim climatic variables among sampled populations indicated that invasive Japanese populations generally experience wetter summers and drier winters than native US populations (PC1, explaining 52.9% of variance). Within each range, our study populations varied across a temperature gradient (PC2, explaining 35.5% of variance), with both ranges spanning similar PC2 coordinates (Figure S1; Tables S1 and S2).

Within each population, we collected seeds from ~50 maternal plants, which grew at least 10 m apart in the field to minimize the probability of sampling the same genetic individual multiple times. We germinated ~10 seeds per maternal plant in a common greenhouse environment (temperature at 18–30°C and relative humidity at 60%) at Monash University, Victoria, Australia, and grew a single individual per maternal family in 12‐cm pots with potting mix with Osmocote. Each population resulted in 18–35 parental (P_1_) individuals (Table S1).

P_1_ individuals from each population were split into groups of five individuals (4–7 replicated groups per population) and crossed within a group using a partial diallel design with reciprocals but no selfing (Lynch & Walsh, 1998). Prior to the anthesis, we bagged branches of inflorescence (~ 5 cm long) with nylon bags to avoid accidental pollen transfer. When >50% of the flowers on a branch had opened, we removed the branch of the sire plant, and rubbed it against an intact branch on a dam plant. Each plant sired four other individuals within the group and received pollen from the four. We also made a self‐pollination cross as a negative control (because *S. altissima* is a self‐incompatible species, self‐crosses did not produce any viable seeds). This resulted in total of 760 crosses (20 crosses per group × 38 groups) across the populations.

#### 2.2.2 Common garden experiment

In 2017, three viable achenes from each cross were selected, and photographed under a dissecting scope for later examination of achene size (see “Seed dispersal ability” below). Seeds of the F_1_ generation were germinated as above, and two seedlings per cross were transplanted to individual 10‐cm diameter pots. A lack of germination from some crosses resulted in a total of 1324 experimental individuals. Potted plants were placed in evenly spaced trays of 17 pots, across six benches (“blocks”) in the greenhouse as above. Trays were rotated weekly to mitigate effects of microclimate within the greenhouse until flowering.

### 2.3 Trait measurements

We measured 10 traits that are thought to mediate plant invasiveness, including growth rate, maximum stem height, SLA, leaf mass, rhizome mass, inflorescence mass, days to first flower, flowering duration, root polyacetylene concentration, and seed size.

#### 2.3.1 Growth, morphology, and phenology

We estimated the relative growth rate of individual plants by measuring height on week 8 and week 10 after transplanting, which corresponded with the period of rapid vertical growth. Relative growth rate was determined as ([height at week 10 – week 8]/14 days). In week 15, we collected three fully expanded leaves from each plant at the height of 60 cm above the base. Each set of three leaves was scanned together, dried for 48 h at 50°C and weighed. Leaf area was measured using ImageJ (version 1.51), and the SLA was calculated as [fresh leaf area/dry mass].

To estimate flower onset and flowering duration, we checked plants daily during the flowering period (week 14 through 22) and marked the dates we observed the first flower and the last senesced flower. Flowering duration for each plant was calculated as days from flowering onset to final day of flowering. We harvested plant biomass between weeks 22 and 25 as individual plants finished flowering. Aboveground biomass was separately harvested for leaves, stems, inflorescence, and ramets. We used inflorescence mass as a proxy for seed production (Root, 1996). We were unable to directly estimate seed production because *S. altissima* is an insect‐pollinated, self‐incompatible species, and does not naturally set seeds in the greenhouse. Belowground rhizomes were harvested by removing roots and washed in water. All harvested samples were dried for 48 h at 50°C before weighing.

#### 2.3.2 Polyacetylene analysis

Root samples were collected for polyacetylene analysis in week 15 by removing a subsample of root tissues from each plant. Root samples were flash frozen in liquid nitrogen and stored in −80°C for later analysis. Following Uesugi et al. (2019), approximately 200 mg fresh weight of root tissue per sample was crushed with mortar and pestle in liquid nitrogen, sonicated in extraction buffer (1 mL of 90% methanol) for 6 min, and left in the dark at room temperature for 24 h. Samples were centrifuged, and 0.5 mL aliquot was filtered with 0.45 μm syringe filter. The samples were analysed with high‐performance liquid chromatography (HPLC) at Monash University using Agilent Infinity 1260 equipped with C18 reserve‐phase column (EC‐C18, 2.7 μm, 150 × 3.0 mm). The elution system consisting of solvents (A) 0.25% H_3_PO_4_ in water (pH 2.2) and (B) acetonitrole was: 0–5 min, 0–20% of B; 5–25 min, 20%–95% of B and 25–30 min, 95% of B, with a flow rate of 0.5 mL min^−1^ and injection volume of 2 μL. Peaks of polyacetylene compounds were identified using UV spectra and quantified at 254 nm (Uesugi et al., 2019).

### 2.4 Seed dispersal ability

Dispersal ability of wind‐dispersed seeds increases with the time they spend airborne (i.e., longer time taken to drop to the ground; Cody & Overton, 1996; Tabassum & Bonser, 2017). To test if seed morphology correlates with drop time, we conducted a separate experiment, where we measured the drop time, achene size and pappus length for 60 randomly selected seed samples. We measured the time taken for a seed to fall a 50 cm distance down a glass cylinder (10 cm diameter) by videotaping and analysing the footage with BORIS software (Friard & Gamba, 2016). Each seed sample was dropped twice, and the average was used for subsequent analysis. The seed was then photographed under dissecting scope, and the achene area (hereafter referred as “seed size” for simplicity) and the maximum pappus length were estimated using ImageJ.

Seed size and pappus length were positively correlated (linear regression: *coefficient* = 0.56, *t* = 4.7, *p* < 0.0001). A multiple regression analysis with model selection using partial *F*‐test showed that seed size alone was the best predictor of seed drop time (*coefficient* = −0.75, *t* = 2.8, *p* = 0.006, *R* ^2^ = 0.14). Thus, we used seed size as a proxy for seed dispersal ability (i.e., smaller seed size increases seed dispersal ability) in the subsequent analyses.

Seed size of plants used in the common garden experiment was estimated using the photographs taken prior to seed germination (see above) using ImageJ. For each cross family, the size of the three largest seeds out of five were measured. Because seeds were bulk germinated for each maternal family, we could not directly link the seed measurement with a specific individual plant sample used in the experiment.

### 2.5 Statistical analyses

All statistical analyses were conducted using R in the RStudio environment (version 3.4.3) (R Core Team, 2021).

#### 2.5.1 Divergence in multivariate invasive phenotypes

We tested for overall trait differences between native and invasive populations using a multivariate linear mixed model, fitted with the *lmer* function of the *lme4* package (Bates et al., 2014). To include seed size in the multivariate model, we used the family means for all trait values in this analysis. The response variables included standardized values (scaled to mean = 0, standard deviation = 1) of 10 focal traits. We used trait, range (native or introduced), and their interactions as fixed effects, and dam and sire as random effects. In this model, we also included source populations' climatic variables representing variation in temperature within each range (PC2 in Tables S1 and S2) as a fixed covariate to control for clinal divergence within each range (Colautti et al., 2009; Woods & Sultan, 2022). In a separate model, we tested variability among populations within each range by modelling as above, but populations as nested within a range. PC2 of source populations was removed from this model, as the values were identical for all individuals within a population. The statistical significance of fixed effects was evaluated using Wald *χ* ^2^ tests.

All interaction terms that included trait effect were significant (see Section 3), indicating that traits were differentially affected by plant origin. Thus, we conducted univariate analyses with linear mixed models as above with range and PC2 as fixed effects, and dam and sire as random effect. We then contrasted populations in univariate models, where population was modelled as nested within a range as a fixed effect, and conducted post hoc tests using the *emmeans* function (*emmeans* package).

#### 2.5.2 Estimation of univariate genetic variances

To explore the amount of genetic variance in each of the 10 focal traits within each population, we first conducted univariate analyses by fitting a linear mixed model with ASReml‐R (Butler et al., 2009). Data were scaled to mean = 0, and standard deviation = 1 prior to analysis (Bolstad et al., 2014). Additive genetic variance in each trait was estimated from pedigree information (i.e., parental identities in the crosses). We also included family identities (where offspring of reciprocal crosses belong to the same family) to estimate nonadditive genetic variance, which includes variance due to dominance and epistasis (Lynch & Walsh, 1998). Block (i.e., greenhouse benches) was included as a fixed effect to control for variability in greenhouse environment.

#### 2.5.3 Correlation between the genetic variance and the magnitude of trait divergence

We tested if the traits that harbour more additive genetic variance (*V*_A_) diverged more between native and invasive ranges (Opedal et al., 2023). The magnitude of trait divergence was estimated as the standardized range effect for each trait from the above analysis (“Divergence in multivariate invasive phenotypes”). The amount of *V*_A_ for each trait was determined as the median of the mean *V*_A_ for the six populations calculated above (“Estimation of univariate genetic variances”). We explored the relationship between divergence and *V*_A_ using a linear regression analysis.

#### 2.5.4 Estimation of genetic (co)variances among invasive traits

Genetic covariances among traits can either constrain or facilitate trait divergence between the ranges. Our limited sample sizes for each population precluded us from examining the multivariate genetic (co)variance structure for all 10 traits, thus we used a subset of traits to examine genetic variances in a multivariate sense. We selected three traits representing invasive strategies—leaf mass representing exploitative competition, polyacetylene concentration for interference competition, and seed size for dispersal ability—to test whether genetic covariances among these traits would constrain evolution of invasion syndrome. These traits showed significant or marginal levels of additive genetic variation in all six populations with univariate analyses (Figure 2), allowing us to estimate genetic covariances among them.

We generated genetic (co)variance matrices (***G***‐matrices) for each of the six populations in a Bayesian framework using an animal model with the *MCMCglmm* package (Hadfield, 2010). As in the univariate analysis with ASReml‐R, random effects included pedigree information and family identities, and block effect was modelled as a fixed effect. The model in matrix form was:

$$y\sim \mathrm{XB}+Z_{a}\sigma_{a}^{2}+Z_{\mathrm{na}}\sigma_{\mathrm{na}}^{2}+\varepsilon ,$$

where *y* is the vector of observations on multivariate traits, X is the design matrix for the fixed effect (B) of blocks within the greenhouse, $Z_{a}$ and $Z_{\mathrm{na}}$ are the design matrices for the random effects estimating additive genetic variance ($\sigma_{a}^{2}$; from the pedigree information) and nonadditive genetic variance ($\sigma_{\mathrm{na}}^{2}$; modelled as “family identity” as above), respectively. The residual (ε) is an unstructured covariance matrix containing the variances of (and covariances between) leaf mass and polyacetylenes, but because we were unable to assign seed size to individual plants (seeds were germinated in bulk for each maternal family), we fixed the covariance between the seed size and all other traits to zero.

We used half‐Cauchy distributions with weakly informative priors based on phenotypic variances of the three traits. Posterior distributions were sampled from 3,150,000 Markov chain Monte Carlo (MCMC) iterations sampled every 3000 iterations after an initial burn‐in of 150,000 iterations. We checked convergence from plots of traces and posterior distributions, and calculated autocorrelations between samples (all were below the recommended level of 0.1, yielding an effective sample size close to 1000 for each parameter). We used 1000 MCMC samples to estimate the mean and 90% HPD intervals for additive genetic covariances of the three traits for each population.

#### 2.5.5 Does genetic covariances constrain evolution in an invasive range?

The theory of resource allocation trade‐offs predicts that evolution of invasion syndrome may be constrained by negative genetic correlations among invasive traits in the introduced range. In contrast, their positive genetic correlations would facilitate concurrent evolution of invasive phenotypes. We tested how the genetic covariance structure might affect population's “evolvability”—an evolutionary trajectory of a population relative to the direction favored by selection (Hansen & Houle, 2008) in the invasive range of *S. altissima* (hereafter, “novel selection”). A relative size of evolvability estimated with an observed full ***G*** matrix, over evolvability estimated with a partial ***G*** matrix without genetic covariances (*R*‐score), would indicate the contribution of covariances on multivariate trait evolution (Agrawal & Stinchcombe, 2009).

Following Hansen and Houle (2008), evolvability e in the direction of selection gradients β can be calculated as:

$$e\left(\beta \right)=\frac{\left\|∆\bar{z}\right\|\cos\left(\theta \right)}{\left\|\beta \right\|}$$

where θ is the angle between the vector representing a selection gradient, β, and the evolutionary response, i.e., $∆\bar{z}=G\times \beta$. Based on the observed pattern of phenotypic divergence in the focal traits between native and invasive *S. altissima* populations, we assumed that novel selection would favor increased values of leaf mass and polyacetylene concentration, and decreased values of seed size that enhance dispersal ability when smaller. Because the novel selection gradients, $\beta_{\text{novel}}$, are unknown in this system, we simulated them by randomly generating a selection gradient for each trait, while setting their signs corresponding to the above expectations (Hangartner et al., 2020). To estimate the uncertainty in evolvability estimates, we used 1000 MCMC samples of ***G*** in each population and $\beta_{\text{novel}}$ generated for each of the ***G*** samples. The metric *R* (Agrawal & Stinchcombe, 2009) was calculated as: [(evolvability calculated with full ***G*** matrix) / (evolvability with all off‐diagonal elements in ***G*** set to zero)]. *R* < 1 implies that covariances constrain the evolution of traits in the direction of selection, whereas *R* > 1 implies that covariances facilitate evolution. We calculated 1000 samples of *R* values and tested against 1 using 90% HPD intervals.

#### 2.5.6 Estimation of genetic covariances among reproductive traits

Examining the relationship between *V*_A_ and trait divergence revealed that rhizome mass and seed size each had ample *V*_A_ within each population but showed relatively low magnitude of trait divergence between the ranges (see the Section 3, Figure 3). As a post hoc test, we explored the hypothesis that evolution of rhizome mass and seed size may be constrained by genetic trade‐offs between other reproductive traits, such as days to first flower. We selected flowering phenology as a trait representing sexual reproduction, as later flowering plants tend to achieve larger aboveground biomass and produce more inflorescence (and are therefore assumed to produce more seeds) than early flowering plants (see Section 3). The flowering trait also had high *V*_A_ within each population (except for Utsunomiya), allowing us to estimate co‐variances with rhizome mass and seed size. The same analyses of genetic covariances and *R*‐scores for this set of traits were conducted as above.

## 3 RESULTS

### 3.1 Divergence in multivariate invasive phenotypes

The multivariate linear mixed model detected significant effects of range (*χ* ^2^ = 102.5, *df* = 1, *p* < 0.0001) and range × trait interaction (*χ* ^2^ = 574.0, *df* = 18, *p* < 0.0001) on overall *S. altissima* phenotypes (Figure S2). When traits were compared among populations, we found a nonsignificant effect of range (*χ* ^2^ = 1.93, *df* = 1, *p* = 0.16), but significant population effect (*χ* ^2^ = 54.6, *df* = 4, *p <* 0.0001), range × trait interaction (*χ* ^2^ = 191.6, *df* = 9, *p <* 0.0001) and population × trait interaction (*χ* ^2^ = 364.8, *df* = 36, *p <* 0.0001). The significant interaction between trait and plant origin (range or population) in both tests indicate that our focal traits are differentially affected by plant origin.

**FIGURE 1:**
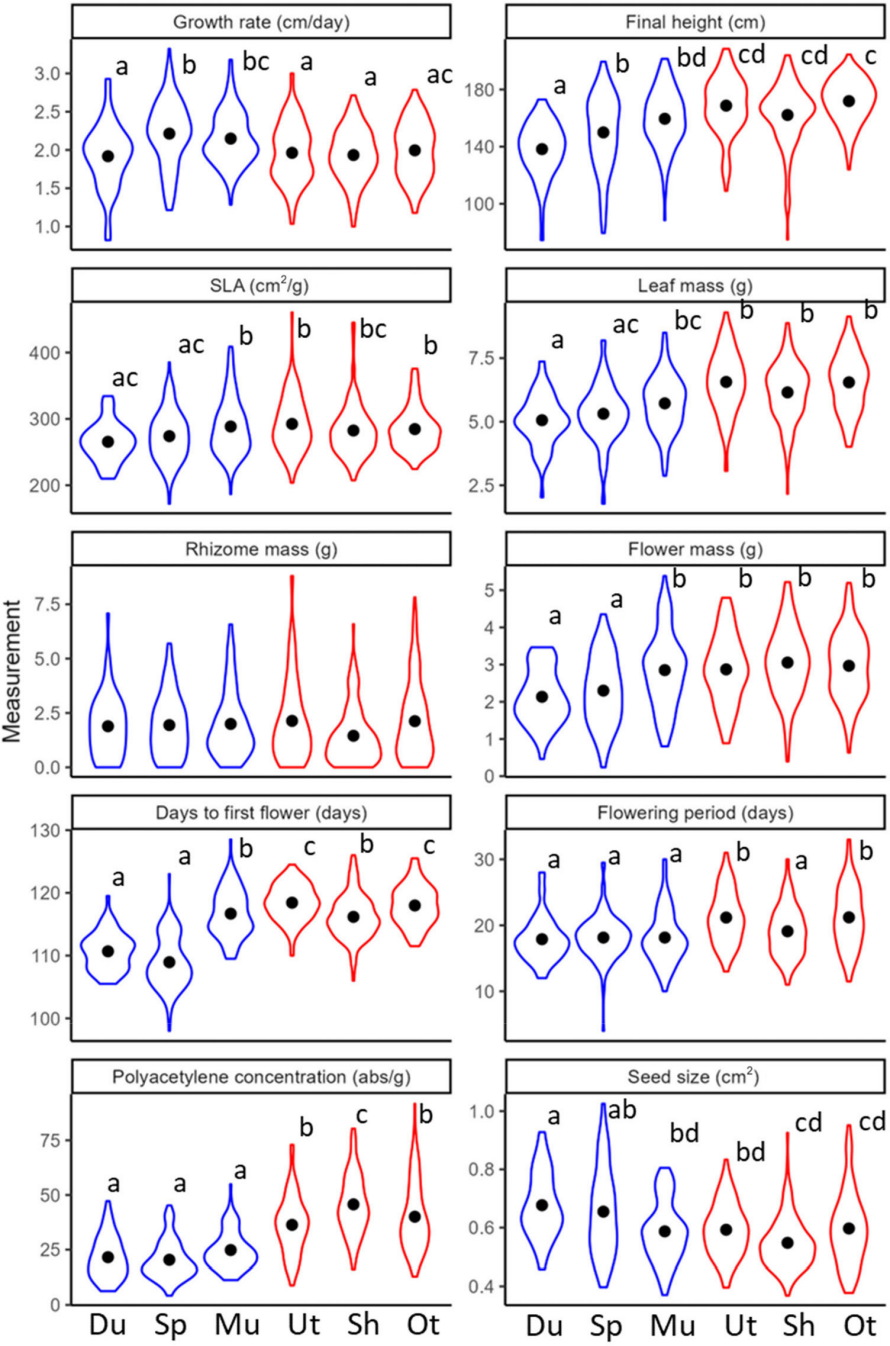
Phenotypic divergence in 10 plant traits across native populations in blue (Du: Durham, Sp: Spartanburg, and Mu: Murrells Inlet) and invasive populations in red (Ut: Utsunomiya, Sh: Shizuoka, Ot: Otsu). Black dots indicate the population means, and letters above violin plots indicate statistical significance with post hoc tests.

Univariate analyses revealed that, in general, plants from the invasive range grew more slowly at a seedling stage but achieved greater final height, leaf biomass and inflorescence mass compared to plants from the native range (Table 1; Figure 1). The onset of flowering was delayed in the invasive range (i.e., longer “days to first flower”), but the duration of flowering period was longer. Plants from the invasive range also produced more allelopathic polyacetylenes in roots, made smaller seeds, but showed no difference in rhizome mass compared to the natives. The temperature gradient (PC2) of source population had positive effects on inflorescence mass, days to first flower, and polyacetylene concentrations, whereas it had a negative effect on flowering period and seed size (Table 1).

**TABLE 1:** Results of univariate analyses testing for the effects of range (invasive Japanese vs. native US populations) and temperature gradient (PC2 in Table S2) on the focal trait values.

	Range			Temperature gradient		
	Coeff.	$\chi^{2}$	*p*	Coeff.	$\chi^{2}$	*p*
Growth rate	**−0.22**	**20.3**	**<0.000**	0.01	0.0	0.840
Height	**0.35**	**50.8**	**<0.000**	0.06	1.5	0.218
SLA	0.11	6.3	0.012	0.04	0.9	0.352
Leaf mass	**0.39**	**72.7**	**<0.000**	0.01	0.1	0.801
Rhizome mass	−0.06	1.4	0.237	−0.08	2.9	0.090
Inflorescence mass	**0.26**	**32.0**	**<0.000**	**0.18**	**15.9**	**<0.000**
Days to first flower	**0.53**	**119.6**	**<0.000**	**0.19**	**15.1**	**<0.000**
Flowering period	**0.28**	**39.5**	**<0.000**	**−0.12**	**7.0**	**0.008**
Polyacetylene []	**0.60**	**211.2**	**<0.000**	**0.19**	**22.1**	**<0.000**
Seed size	**−0.27**	**18.7**	**<0.000**	**−0.23**	**14.1**	**<0.000**

*Note*: Coefficients estimate for the effect of range indicates the change in mean‐standardized trait values of invasive populations relative to native populations. Bold letters indicate statistical significance after Bonferroni correction for 10 traits (α<0.005).

While overall differences between native and invasive ranges were found, we observed variability in trait values among populations within each range (Figure 1). In general, plants from Murrells Inlet, the southern‐most population in the native range, showed similar phenotypes to plants from invasive populations (Figure 1). A clear‐cut divergence between the native and invasive populations was found only for polyacetylene concentrations, where all invasive populations produced more root polyacetylenes than all native populations.

### 3.2 Univariate estimates of additive genetic variance

**FIGURE 2:**
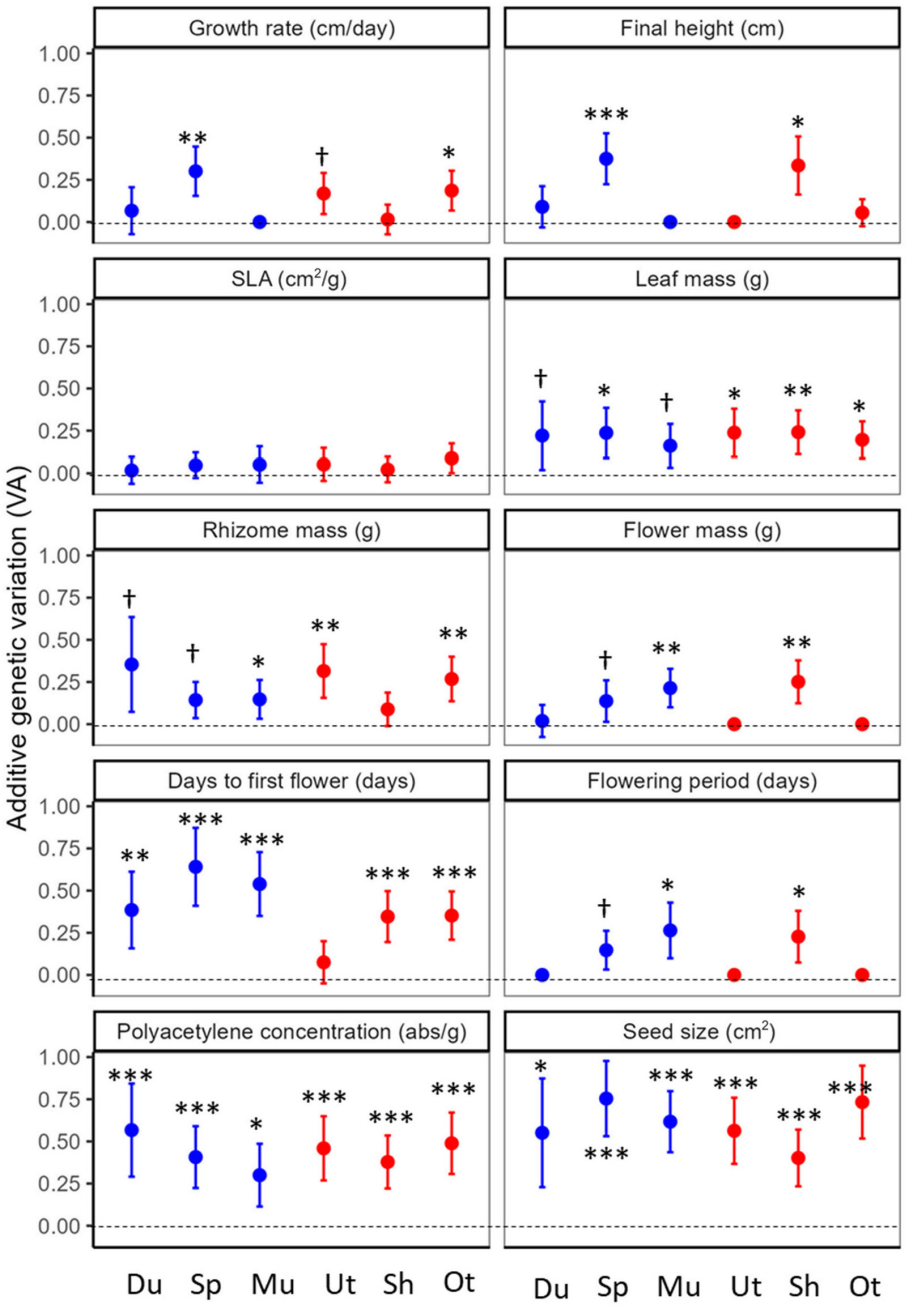
Univariate estimates of additive genetic variance for 10 plant traits across native populations (Du: Durham, Sp: Spartanburg, and Mu: Murrells Inlet) and invasive populations (Ut: Utsunomiya, Sh: Shizuoka, Ot: Otsu). Error bars indicate standard errors of the estimates, and asterisks indicate statistical significance tested using log‐likelihood ratio tests with ASReml‐R: ^†^ *p* < 0.1, *p < 0.05, **p < 0.01, ***p < 0.001.

Populations differed in the amount of additive genetic variance (*V*_A_) for the 10 traits, with some traits showing significant *V*_A_ in some populations, but not in the other populations (Figure 2). We found relatively large and significant (or nearly so) *V*_A_ across all populations for leaf mass, polyacetylene concentrations, and seed size, accounting for between 16%–24%, 30%–57%, and 40%–75% of phenotypic variance, respectively. In contrast, *V*_A_ for SLA was small (<10%) and nonsignificant for all populations. While we observed variation among populations, we found no systematic difference in *V*_A_ between native and invasive ranges in any of the traits (Figure 2). Nonadditive genetic variation (i.e., dominance and epistasis) contributed little to the total phenotypic variation, accounting for <10% of variation in most of trait‐population combinations.

### 3.3 Correlation between the genetic variance and the magnitude of trait divergence

**FIGURE 3:**
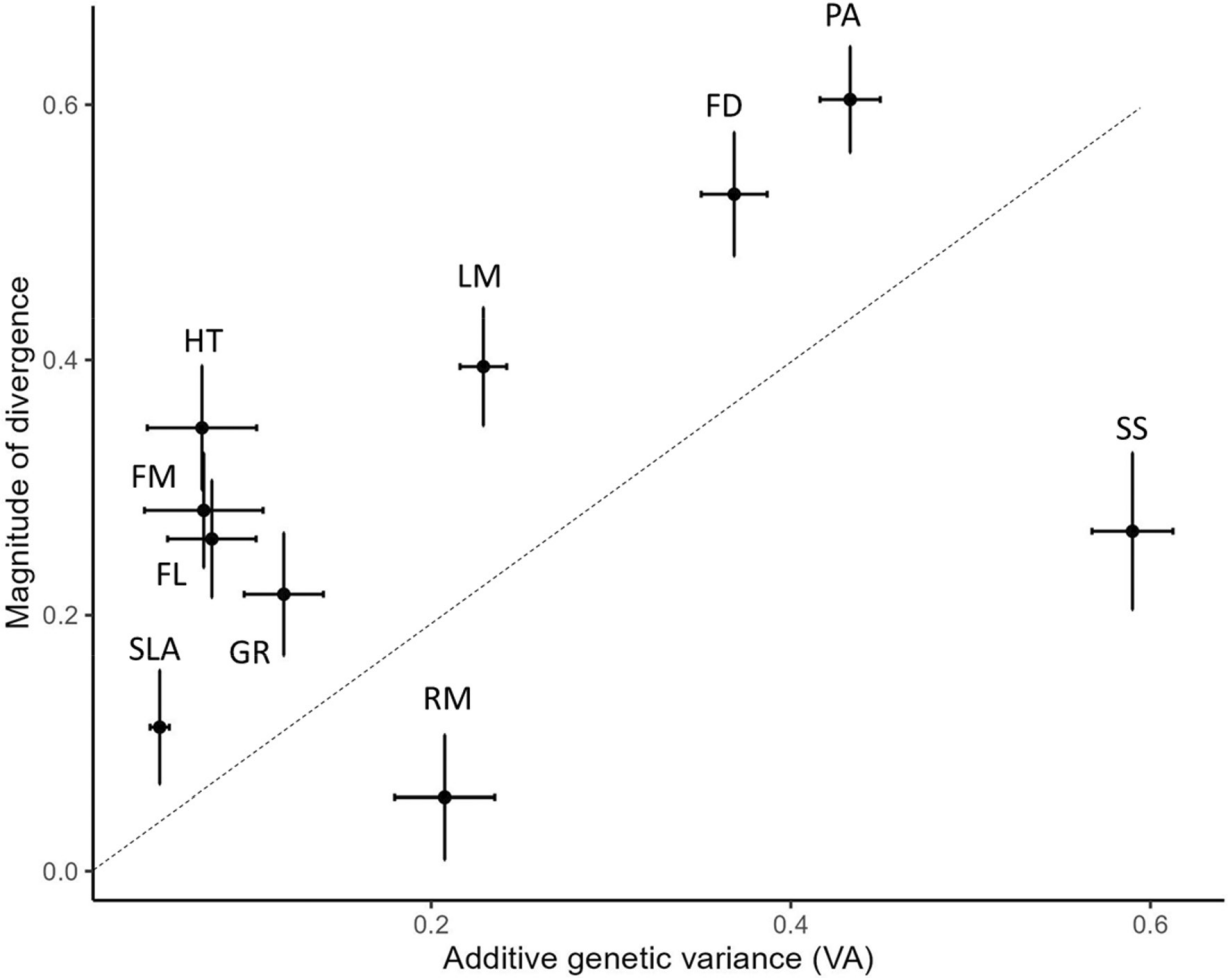
Relationship between the mean standardized magnitude of trait divergence between native and invasive ranges (in absolute values) and the mean‐standardized additive genetic variance (*V*_A_) for 10 focal traits: GR = growth rate, HT = final height, SLA = specific leaf area, LM = leaf mass, RM = rhizome mass, FM = inflorescence mass, FD = days to first flower, FL = flowering duration, PA = polyacetylene concentration, and SS = seed size. The trait divergence was estimated using a liner mixed model, and the vertical error bars indicate standard errors calculated in the *lmer* model. For each trait, median values of *V*_A_ were calculated from the estimated *V*_A_ values of the six populations. Horizontal error bars indicate standard errors. Dotted line indicates 1:1 ratio.

Across the 10 focal traits, the magnitude of trait divergence between native and invasive ranges did not correlate with the *V*_A_ (median of six populations, *t* = 1.7, *p* = 0.14; Figure 3). Polyacetylene concentration and the days to first flower (PA and FD in Figure 3, respectively) both showed high divergence and *V*_A_, while SLA showed the opposite end of the spectrum. Seed size and rhizome mass, in contrast, showed relatively low divergence for the observed amount of *V*_A_ (SS and RM in Figure 3, respectively), suggesting that evolution of seed size and rhizome mass could be constrained by other factors besides *V*_A_ per se. When we removed seed size and rhizome mass from the analysis, we found a strong relationship between divergence and *V*_A_ (*t* = 5.3, *p* = 0.002), suggesting that these two traits are outliers of the trend.

### 3.4 Genetic covariances among main invasive traits

In multivariate analysis, we found relatively small additive genetic covariances (Cov_A_) among leaf mass, polyacetylene concentration, and seed size in all populations, with 90% HPD intervals overlapping with zero in all contrasts (Table 2a). While not significantly different from zero, the posterior means of Cov_A_ between leaf mass and polyacetylenes, and seed size and polyacetylenes, were positive in five out of six populations. We observed no systematic differences between native vs. invasive populations in the signs or magnitude of Cov_A_ (Table 2a).

**TABLE 2:** Estimated posterior means of pair‐wise additive genetic covariances for the (a) competitive traits (leaf mass, polyacetylene concentration, and seed size) and (b) reproductive traits (days to first flower, rhizome mass, and the seed size) using a Bayesian approach.

(a) Competitive traits			
	Leaf mass vs. polyacetylenes	Leaf mass vs. seed size	Polyacetylene vs. seed size
Durham	0.10 (−0.1/0.33)	0.04 (−0.26/0.32)	0.20 (−0.17/0.6)
Spartanburg	0.15 (−0.04/0.32)	0 (−0.17/0.16)	−0.09 (−0.31/0.11)
Murrells Inlet	0.05 (−0.06/0.17)	−0.12 (−0.3/0.05)	0.02 (−0.15/0.22)
Utsunomiya	−0.01 (−0.18/0.14)	−0.17 (−0.38/0.05)	0.12 (−0.12/0.38)
Shizuoka	0.12 (−0.05/0.32)	0.20 (−0.01/0.41)	0.22 (−0.01/0.46)
Otsu	0.04 (−0.08/0.18)	−0.06 (−0.22/0.07)	0.04 (−0.22/0.3)

(b) Reproductive traits			
	Rhizome mass vs. days to first flower	Seed size vs. days to first flower	Rhizome mass vs. seed size
Durham	0.09 (−0.13/0.36)	−0.1 (−0.54/0.28)	−0.15 (−0.57/0.17)
Spartanburg	−0.02 (−0.14/0.12)	−0.17 (−0.41/0.06)	−0.01 (−0.16/0.09)
Murrells Inlet	0.11 (−0.02/0.26)	**−0.35 (−0.62/‐0.08)**	−0.07 (−0.23/0.09)
Utsunomiya	0.01 (−0.08/0.12)	−0.07 (−0.25/0.07)	−0.04 (−0.27/0.19)
Shizuoka	0 (−0.08/0.08)	−0.08 (−0.28/0.13)	−0.03 (−0.14/0.05)
Otsu	0.03 (−0.16/0.18)	−0.04 (−0.25/0.2)	−0.16 (−0.38/0.06)

*Note*: Values in () indicate upper and lower 90% HPD intervals.

Bold values indicate no overlap of 90% HPD intervals with zero.

**FIGURE 4:**
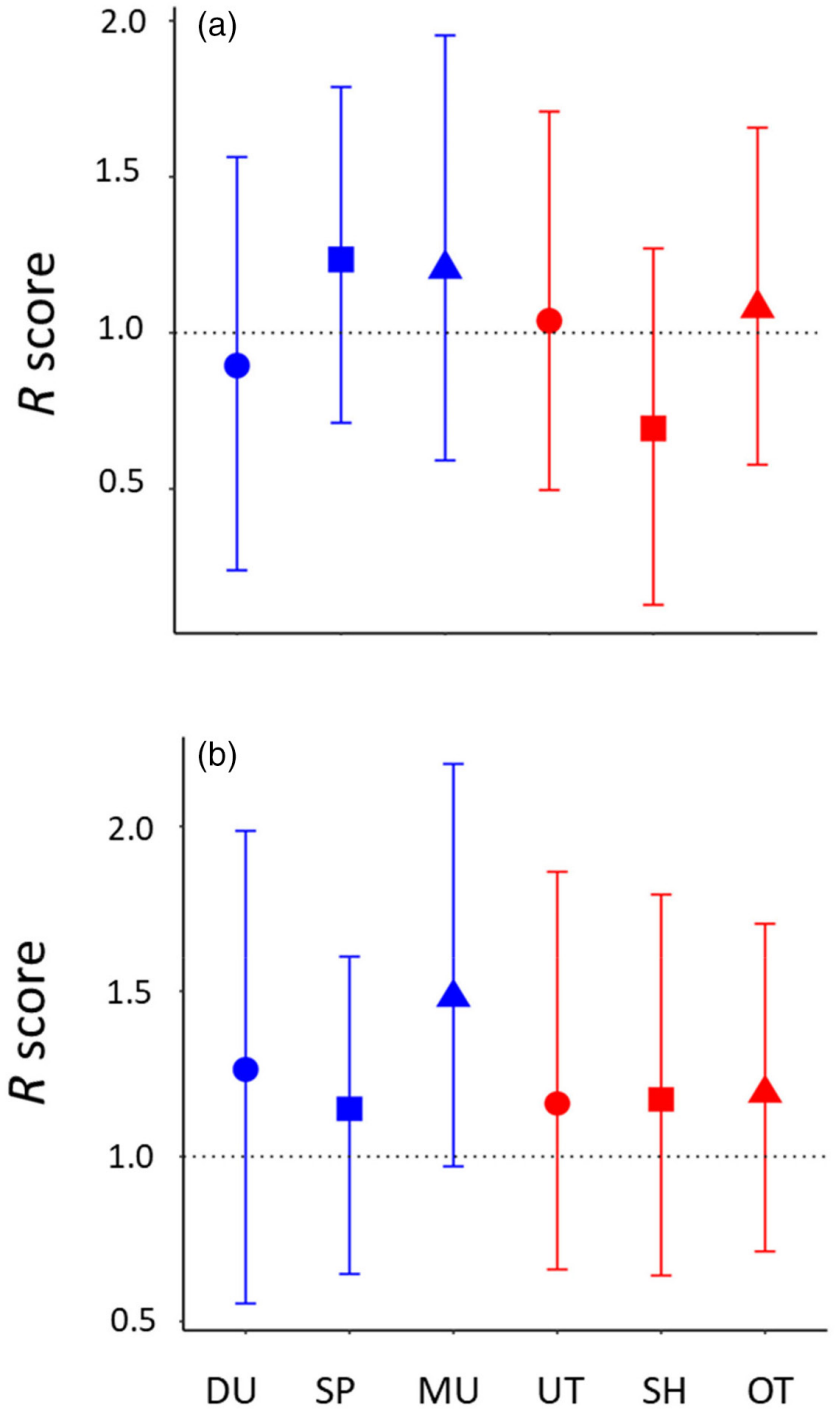
Effect of genetic covariances on the evolvability of populations summarised as *R*‐score. (a) for invasive traits (leaf mass, polyacetylenes & seed size), and (b) for reproductive traits (days to first flower, rhizome mass & seed size). Blue indicates native populations (Du: Durham, Sp: Spartanburg, and Mu: Murrells Inlet) and red indicate invasive populations (Ut: Utsunomiya, Sh: Shizuoka, Ot: Otsu).

To explore whether covariances among the three invasive traits would constrain evolution of each trait, we calculated *R* metric. In all populations, *R* scores did not significantly deviate from 1 (HPD intervals overlapped 1, Figure 4a), indicating that genetic covariances among three traits neither constrain nor accelerate their evolution. We found no differences in *R* score among populations.

### 3.5 Genetic trade‐offs among reproductive traits

Similar to the previous set of traits, we found relatively small Cov_A_ among rhizome mass, seed size and days to first flowering (Table 2b). All 90% HPD intervals overlapped with zero, except for negative covariance between days to first flower and seed size in the Murrells Inlet population. Although not significant, on average, rhizome mass positively covaried with days to first flower in five out of six populations. The mean covariances were negative between seed size and flowering day, and seed size and rhizome mass in all populations. The mean R scores were above 1 in all populations, but HPD intervals strongly overlapped with 1 (Figure 4b). The results suggest that genetic covariances among reproductive traits do not constrain concerted evolution of these traits but, if anything, may facilitate concerted evolution of increased sexual and asexual reproduction.

## 4 DISCUSSION

### 4.1 Evolution of the invasion syndrome

We found an overall shift in functional traits between native US and invasive Japanese populations of *S. altissima*. As expected under the EICA hypothesis (Blossey & Notzold, 1995), invasive populations generally had higher aboveground biomass, indicating a superior ability to exploit limited light resources (Rotter & Holeski, 2018). But invasive populations did not achieve greater final biomass by growing faster, but rather by delaying flowering, thereby extending the period for vegetative growth. A larger biomass, in turn, resulted in higher inflorescence mass, which indicates greater seed production in the invasive populations. Such life history trade‐offs between final biomass and reproduction are commonly observed in plants adapting to varying environments (Colautti & Barrett, 2011; van Boheemen et al., 2019; Woods & Sultan, 2022). Consistent with the novel weapons hypothesis (Callaway & Ridenour, 2004), we also observed increased polyacetylene production in invasive *S. altissima*, suggesting that enhanced allelopathy had evolved in the invasive range. Finally, we found that invasive populations generally produced greater quantities of seeds, which are smaller (thereby staying airborne longer) and are released from a greater height, suggesting their superior dispersal ability (Thomson et al., 2011; Williams et al., 2016). Our estimates of sexual reproduction in nature could be biased if invasive and native populations differ in pollinator attraction and fertilization success, as we used inflorescence mass as a proxy for seed production. Our approach may have underestimated the difference in seed set between the ranges: larger inflorescence of Japanese populations is likely to attract more generalist pollinators in the field (Ikemoto et al., 2017) than US populations, and previous hand‐pollination trials in a controlled greenhouse environment found slightly higher proportions of seed‐set in Japanese populations than in US populations (Uesugi et al., 2020).

The observed divergence in plant phenotypes is likely to be the result of adaptation to novel competitive environments in the introduced range. To contrast invasive populations with its native origin, we chose native populations from the south‐eastern US, where Japanese populations are thought to have originated (Sakata et al., 2015). However, we note that traits varied among native populations, and that the southern‐most population in the native range (Murrells Inlet) tended to exhibit similar phenotypes as to Japanese populations. While we sampled a limited number of populations to make the quantitative genetic analyses feasible, a previous study that contrasted US and Japanese *S. altissima* populations across broader latitudinal ranges (*n* = 9) showed similar patterns of divergence in plant biomass and polyacetylene concentrations (Uesugi et al., 2020). We also contrasted plant traits in a common garden, using seeds from maternal plants that were also grown in a common environment. Thus, maternal effects are likely to be negligible. Finally, we statistically controlled for the effect of environmental gradient on trait variation. Because local adaptation along a climatic gradient can often lead to trait divergence within each range, failure to control for it could lead to erroneous conclusions about adaptation to a novel range per se (Colautti et al., 2009). We showed that the trait differences between native and invasive ranges remained significant after controlling for the source populations' temperature gradient (PC2) that varied among populations within each range.

### 4.2 Evolution of invasive traits is fuelled by abundant additive genetic variations

Adaptive evolution of invasive traits requires that introduced populations maintain sufficient additive genetic variation (*V*_A_) for these traits for selection to act on. Introduced populations are expected to have reduced genetic variability due to founder effects, and strong natural selection for invasive traits in the introduced range may erode genetic variation for these traits (Blows & Walsh, 2009; Shaw et al., 1995). In contrast to these expectations, we found substantial *V*_A_ in many of the 10 traits within invasive Japanese populations with no systematic difference from native populations. Our results suggest that the current invasive *S. altissima* populations do not have reduced genetic variations—similar to other studies that contrasted quantitative genetic variation in plant traits between introduced and native populations (Calsbeek et al., 2011; Franks et al., 2012; McGoey & Stinchcombe, 2021; Sakata et al., 2020). Nonadditive genetic variation (i.e., dominance and epistasis) was negligeable in many cases, in agreement with previous studies that additive genetic variance, which can respond to natural selection, accounts for the majority of genetic variance in complex traits (Hill et al., 2008).

Genetic covariance among traits could also constrain evolution of multiple invasive traits. We predicted genetic trade‐offs among three major invasive traits, but found genetic covariance among them to be generally small across all populations. While not significant, leaf mass and production of polyacetylenes tended to covary positively, suggesting that ability to exploit resources and suppress competitors through allelopathy may go hand in hand. *R* scores (Agrawal & Stinchcombe, 2009) also indicated that genetic covariances among these competitive traits do not constrain their evolution. Thus, we suggest that the “invasion syndrome” in *S. altissima* has evolved in the novel range due to ample genetic variation, and relatively free from genetic constraints. Similar weak facilitative effects of genetic covariances were found in ragweed (*Ambrosia artemisiifolia*), where both native and invasive populations positively covaried in plant biomass, flowering phenology and sexual reproduction, suggesting that the covariance structure may accelerate evolution of invasive phenotypes (McGoey & Stinchcombe, 2021).

### 4.3 Evolution of allelopathy was pronounced in the invasive range

Among studied traits, some diverged more than the others between the ranges. In particular, the production of allelopathic polyacetylenes showed a greater magnitude of divergence between the ranges compared to other traits and was the only trait that clearly differed between the ranges (Figure 1). Multiple, nonmutually exclusive factors could contribute to the enhanced evolutionary increase in polyacetylene production: First, natural selection for allelopathy may be particularly strong in the invasive range because allelochemicals are likely to be more effective against naïve indigenous competitors (Callaway & Ridenour, 2004; Zhang et al., 2021). Second, its evolutionary response to selection may be strong due to large *V*_A_ for the trait within a population (Opedal et al., 2023, Figure 2). Other studies have shown that *V*_A_ for plant secondary metabolites was generally greater than morphological traits in plants (Geber & Griffen, 2003; Johnson et al., 2009). Third, a relaxation of herbivory in the introduced range could drive evolution of increased allelopathy due to alleviation of trade‐offs with defence traits (Blossey & Notzold, 1995). Uesugi and Kessler (2013) found more pronounced evolutionary increase in polyacetylene production compared to other traits after a 12‐year experimental removal of herbivory from *S. altissima*. Finally, a release from herbivory in the invasive range may reshape the genetic covariance structure of secondary metabolites to facilitate evolution of allelopathy in the novel range (Franks et al., 2012; Uesugi et al., 2017). A previous quantitative genetics analysis (Uesugi et al., 2017) suggested that a lack of selection from herbivory exposed negative genetic covariance between polyacetylenes and defense compound productions (Van Noordwijk & De Jong, 1986). Although such a shift in genetic covariance is likely to be transient (Uesugi et al., 2017), it could facilitate a rapid evolution of allelopathy under negative correlational selection that acts against energetically costly defense traits while favoring increased allelopathy (Herms & Mattson, 1992).

A limited number of studies have compared allelopathy between native and invasive ranges so far (Bock et al., 2018; Gruntman et al., 2016; Irimia et al., 2019; Oduor et al., 2020; Sotes et al., 2021; Uesugi et al., 2020; Yuan et al., 2013; Zheng et al., 2015), and they show mixed results. Invasive populations of *S. canadensis* (Yuan et al., 2013) and *Chromolaena odorata* (Zheng et al., 2015) produced more allelopathic compounds than their native counterparts, but the opposite was true for *Impatiens glandulifera* (Gruntman et al., 2016), and no difference was observed for *Helianthus tuberosus* (Bock et al., 2018), *Centaurea solstitialis* (Irima et al., 2019), *Brassica nigra* (Oduor et al., 2020) and *Centaurea melitensis* (Sotes et al., 2021). Our study indicated that evolution of allelopathy may have been particularly important for the widespread invasion of *S. altissima* in Japan, but its relative contribution for plant invasion across plant taxa is yet to be tested (Hierro & Callaway, 2021).

### 4.4 Some traits evolved less than predicted by the amount of genetic variance

Given selection, traits with larger additive genetic variance are expected to diverge more between the ranges (Opedal et al., 2023), but we found a relatively small degree of divergence in seed size and rhizome mass that had relatively large amounts of *V*_A_. We hypothesized that genetic covariance among reproductive traits may constrain the evolution of seed size and rhizome production. Contrary to the expectation, genetic covariances among seed size, rhizome mass and days to first flower were generally small. On average, we found that individuals that flower later tend to produce more rhizomes, indicating that delayed flowering allows plants to build up biomass both above‐ and belowground. Late flowering plants tended to produce smaller seeds, which are likely to disperse farther. Finally, those that produced more rhizomes tended to have smaller seeds. Overall, we found no evidence that genetic covariances among these reproductive traits are constraining their evolution.

Why then, did we observe limited divergence in rhizome production and seed size? We speculate that the strength of natural selection on these traits in the introduced range may have decreased once the populations became well established (Blossey et al., 2021; Lankau et al., 2009). For example, while increased rhizome mass may facilitate initial colonization and establishment in self‐incompatible species, vegetative reproduction could become less important when populations become capable of colonizing new habitats via seed dispersal (Uesugi et al., 2020). Similarly, increased seed dispersal ability can be advantageous at the expanding edge of invasive populations, but the benefit may decrease as populations become well established (Phillips et al., 2006). For instance, a study contrasting seed characteristics along the invasion route in *Senecio inaequidens* (Monty & Mahy, 2010) and *Gladiolus gueinzii* (Tabassum & Leishman, 2018) found reduced plume load (i.e., higher dispersal ability) away from its introduction site. However, as the density of the invader increases, selection should favor larger seeds that may be more successful at establishing in highly competitive environments (Maron et al., 2021). It is worth noting that although *S. altissima* has been established in Japan for more than a century, the seed size has not returned to the level of its native populations. Because *S. altissima* opportunistically spreads by colonizing newly disturbed habitats, maintaining a high dispersal ability may be beneficial even after the invasive populations have become widespread. Observed evolution of dispersal ability may make Japanese *S. altissima* populations particularly “weedy” within the invaded range.

## 5 CONCLUSIONS

We found evidence that an invasion syndrome has evolved in introduced Japanese *S. altissima* populations, making them more competitive and highly dispersive than its native conspecifics. A large amount of additive genetic variation and lack of genetic trade‐offs among these traits in the current invasive populations suggest that the Japanese populations could further respond to selection in the invasive range. Whether invasive traits will continue to evolve in Japanese populations is unknown, as the direction and strength of natural selection could change over time. For instance, the recent introduction of lace bug (*Corythucha marmorata*) into Japan has resulted in a rapid evolution of plant resistance in *S. altissima* against the herbivory (Sakata et al., 2014). In the presence of such herbivores, negative genetic covariance between defensive traits and competitive traits (Uesugi et al., 2017) could hinder further evolution of invasive traits (Gruntman et al., 2016). Similarly, counter‐adaptation to *S. altissima*'s allelopathy by indigenous plant species could also weaken selection for increased allelopathy in the invasive range (Lankau et al., 2009; Rowe & Leger, 2011). Such temporal and spatial variation in natural selection within the introduced range may contribute to the maintenance of genetic variation within invasive plant populations.

## CONFLICT OF INTEREST STATEMENT

The authors declare no conflicts of interest.

## Supporting information


Data S1

## Data Availability

Data are deposited in Dryad (DOI: 10.5061/dryad.n8pk0p34b).
