# Supplementary material for: Evolution of “invasion syndrome” in invasive goldenrod is not constrained by genetic trade‐offs
Source: Evol Appl. 2024 Jun 28;17(7):e13734. doi: 10.1111/eva.13734 (PMC11211922; doi:10.1111/eva.13734)
Supplement: Supplementary file 1 — Data S1 [file EVA-17-e13734-s001.docx]

Supplementary materials

**Table S1**: Locality of source populations of *Solidago altissima* where original seeds were collected from. Sample size indicates the number of F_1_ individuals phenotyped in the common garden experiment, and the values in ( ) indicate number of P_1_ individuals used to create the F_1_ generation. PC1 and 2 give values of the first and second principal component of 19 bioclimatic variables (WorldClim, PCA loadings are shown in Table S2).

|  | Population | State/  Prefecture | Latitude | Longitude | PC1 | PC2 | Sample size |
| --- | --- | --- | --- | --- | --- | --- | --- |
| Native | Durham | North Carolina | 36.031 | -78.978 | -2.48 | 1.93 | 298 (18) |
| (USA) | Spartanburg | South Carolina | 35.003 | -81.973 | -3.85 | 1.06 | 612 (35) |
|  | Murrells In | South Carolina | 33.609 | -79.015 | -2.20 | -3.14 | 575 (35) |
| Invasive | Utsunomiya | Tochigi | 36.514 | 139.947 | 3.23 | 2.97 | 540 (30) |
| (Japan) | Shizuoka | Shizuoka | 34.961 | 138.434 | 2.69 | -3.18 | 638 (35) |
|  | Otsu | Shiga | 34.953 | 135.945 | 2.61 | 0.36 | 638 (35) |

| **Table S2**: Results of PCA analysis of variation in 19 WorldClim climatic variables for populations used in this study. The first and the second principal components explained 88.4% of all variables. PC1 primarily described variation in precipitation, with Japanese populations experiencing wetter summer and drier winter than US populations (see Table S1). PC2 was correlated with variation in temperature parameters. Both US and Japanese populations spanned similar range of PC2 values.   \| Bioclim \| Description \| PC1  (52.9%) \| PC2  (35.5%) \| \| --- \| --- \| --- \| --- \| \| bio1 \| Annual Mean Temperature (C) \| -0.48 \| 0.85 \| \| bio2 \| Mean Diurnal Range \| -0.93 \| -0.34 \| \| bio3 \| Isothermaility \| -0.98 \| -0.04 \| \| bio4 \| Temperature Seasonality (standard deviation x 100) \| 0.40 \| -0.82 \| \| bio5 \| Max Temperature of Warmest Month (C) \| -0.90 \| 0.20 \| \| bio6 \| Min Temperature of Coldest Month (C) \| -0.09 \| 0.97 \| \| bio7 \| Temperature Annual Range (C) \| -0.44 \| -0.89 \| \| bio8 \| Mean Temperature of Wettest Quarter (C) \| 0.48 \| 0.31 \| \| bio9 \| Mean Temperature of Driest Quarter (C) \| -0.94 \| 0.27 \| \| bio10 \| Mean temperature of Warmest quarter (C) \| -0.44 \| 0.83 \| \| bio11 \| Mean Temperature of Coldest Quarter (C) \| -0.40 \| 0.89 \| \| bio12 \| Annual Precipitation (mm) \| 0.57 \| 0.65 \| \| bio13 \| Precipitation of Wettest Month (mm) \| 0.77 \| 0.58 \| \| bio14 \| Precipitation of Driest Month (mm) \| -0.89 \| 0.24 \| \| bio15 \| Precipitation of seasonality \| 0.95 \| 0.17 \| \| bio16 \| Precipitation of Wettest Quarter (mm) \| 0.74 \| 0.62 \| \| bio17 \| Precipitation of Driest Quarter (mm) \| -0.87 \| 0.34 \| \| bio18 \| Precipitation of Warmest Quarter (mm) \| 0.77 \| 0.60 \| \| bio19 \| Precipitation of Coldest Quarter (mm) \| -0.91 \| 0.35 \| |  |  |  |
| --- | --- | --- | --- | --- | --- | --- | --- | --- | --- | --- | --- | --- | --- | --- | --- | --- | --- | --- | --- | --- | --- | --- | --- | --- | --- | --- | --- | --- | --- | --- | --- | --- | --- | --- | --- | --- | --- | --- | --- | --- | --- | --- | --- | --- | --- | --- | --- | --- | --- | --- | --- | --- | --- | --- | --- | --- | --- | --- | --- | --- | --- | --- | --- | --- | --- | --- | --- | --- | --- | --- | --- | --- | --- | --- | --- | --- | --- | --- | --- | --- | --- | --- | --- |
|  |  |  |  |
| **Table S3**: Results of univariate analyses testing for the effects of plant origin and **latitude** on the focal trait values. Coefficients estimate the change in mean-standardized trait values of invasive populations relative to native populations. Bold letters indicate significance after Bonferroni correction for 10 traits ($\alpha<0.005)$.   \|  \| Origin \| \| \| Latitude \| \| \| \| --- \| --- \| --- \| --- \| --- \| --- \| --- \| \|  \| ***Coeff*** \| $\boldsymbol{\chi}^{\boldsymbol{2}}$ \| ***P*** \| ***Coeff*** \| $\boldsymbol{\chi}^{\boldsymbol{2}}$ \| ***P*** \| \| Growth rate \| **-0.18** \| **11.6** \| **0.001** \| -0.09 \| 2.5 \| 0.11 \| \| Height \| **0.42** \| **63.8** \| **<0.0001** \| **-0.19** \| **11.3** \| **0.001** \| \| SLA \| **0.14** \| **8.5** \| **<0.0001** \| -0.08 \| 2.5 \| 0.12 \| \| Leaf mass \| **0.43** \| **71.3** \| **<0.0001** \| -0.09 \| 2.7 \| 0.10 \| \| Rhizome mass \| -0.07 \| 1.4 \| 0.23 \| 0.03 \| 0.3 \| 0.60 \| \| Inflorescence mass \| **0.35** \| **46.5** \| **<0.0001** \| **-0.24** \| **18.8** \| **<0.0001** \| \| Days to first flower \| **0.64** \| **148.0** \| **<0.0001** \| **-0.30** \| **27.3** \| **<0.0001** \| \| Flowering period \| **0.27** \| **27.8** \| **<0.0001** \| 0.05 \| 0.9 \| 0.36 \| \| Polyacetylene \| **0.66** \| **196.9** \| **<0.0001** \| **-0.18** \| **12.0** \| **0.001** \| \| Seed size \| **-0.35** \| **26.4** \| **<0.0001** \| **0.25** \| **11.5** \| **0.001** \| |  |  |  |
| 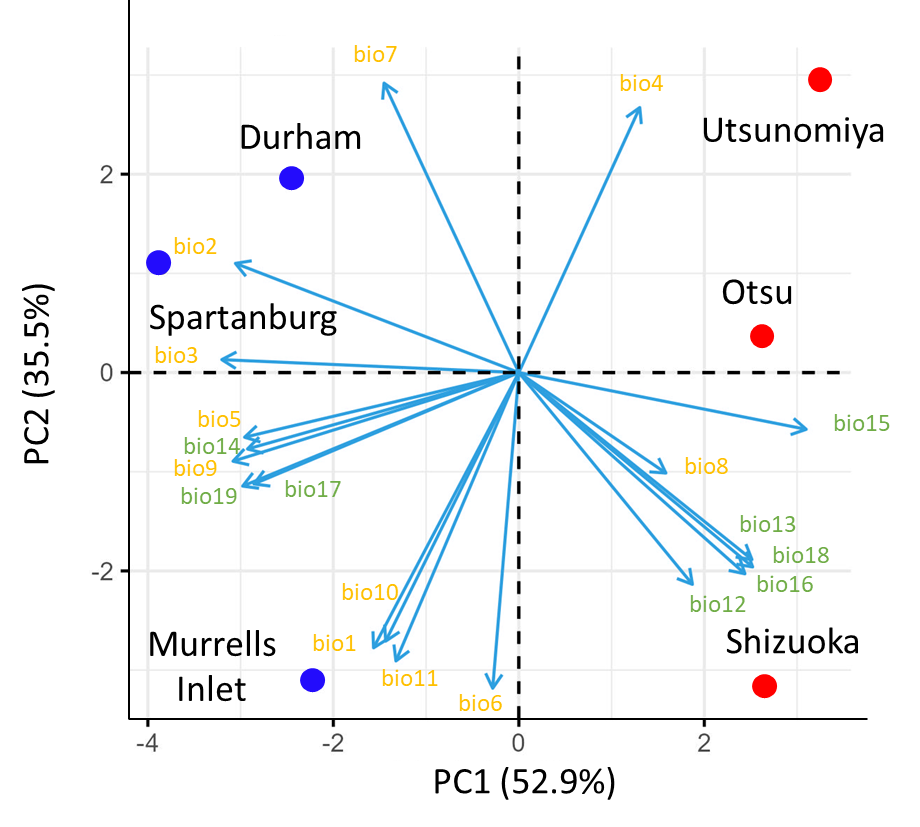  **Figure S1**: PCA plot of 19 WorldClim climatic variables of sampled populations (native US populations in blue and invasive Japanese populations in red). |  |  |  |
|  |  |  |  |
|  |  |  |  |
|  |  |  |  |


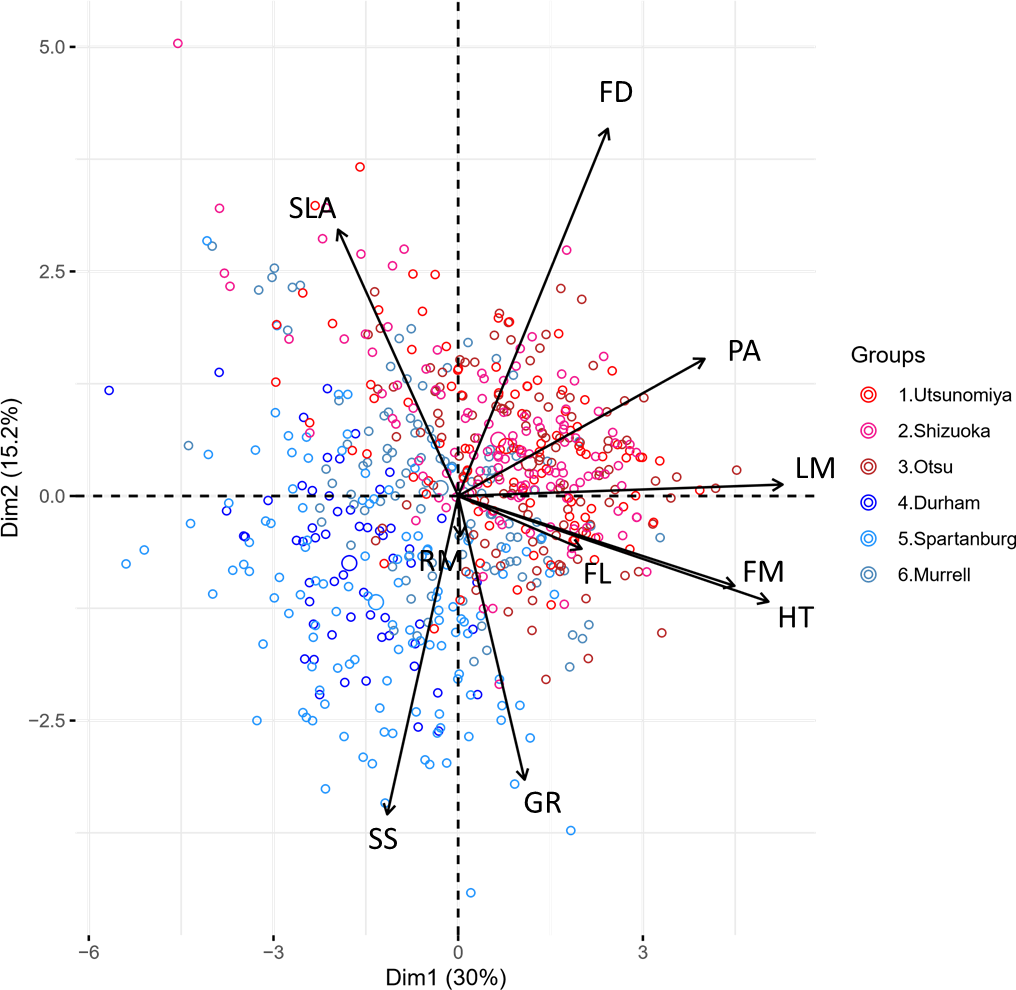


**Figure S2**: Phenotypic trait correlation among 10 focal traits analysed using a principal component analysis (PCA): GR = growth rate, HT = final height, SLA = specific leaf area, LM = leaf mass, RM = rhizome mass, FM = inflorescence mass, FD = days to first flower, FL = flowering duration, PA = polyacetylene concentration, and SS = seed size. Native populations are indicated by blue shades (Durham, Spartanburg, Murrell) and invasive populations by red shades (Utsunomiya, Shizuoka, Otsu).
